# Motivations of children and their parents to participate in drug research: a systematic review

**DOI:** 10.1007/s00431-016-2715-9

**Published:** 2016-04-04

**Authors:** Krista Tromp, C. Michel Zwaan, Suzanne van de Vathorst

**Affiliations:** Department of Medical Ethics and Philosophy of Medicine, Erasmus MC, P.O. box 2040, 3000 CA Rotterdam, The Netherlands; Department of Pediatric Oncology/Hematology, Erasmus MC-Sophia Children’s Hospital, P.O. box 2040, 3000 CA Rotterdam, The Netherlands

**Keywords:** Clinical drug research, Child, Parents, Motivations, Participation, Ethics

## Abstract

Information on motivations for research participation, may enable professionals to better tailor the process of recruitment and informed consent to the perspective of parents and children. Therefore, this systematic review assesses motivating and discouraging factors for children and their parents to decide to participate in clinical drug research. Studies were identified from searches in 6 databases. Two independent reviewers screened and selected relevant articles. Results were aggregated and presented by use of qualitative metasummary. 38 studies fulfilled the selection criteria and were of sufficient quality for inclusion in the qualitative metasummary. Most mentioned motivating factors for parents were: health benefit for child, altruism, trust in research, and relation to researcher. Most mentioned motivating factors for children were: personal health benefit, altruism and increasing comfort. Fear of risks, distrust in research, logistical aspects and disruption of daily life were mentioned most by parents as discouraging factors. Burden and disruption of daily life, feeling like a “guinea pig” and fear of risks were most mentioned as discouraging factors by children.

*Conclusion*: Paying attention to these motivating and discouraging factors of children and their parents during the recruitment and informed consent process in drug research increases the moral and instrumental value of informed consent.

**Table:** 

**What is known:**
• *This systematic review pools the existing empirical literature on motivations of minors and their parents to consent or dissent to participation in clinical drug research*.
• *The most mentioned motivating and discouraging factors for children and their parents to consent to participation in clinical drug research are identified aggregated and presented by use of qualitative metasummary*.
**What is new:**
• *This information can be used to adapt the research protocol, recruitment, and informed consent/assent process to the needs of children and their parents*.

**Electronic supplementary material:**

The online version of this article (doi:10.1007/s00431-016-2715-9) contains supplementary material, which is available to authorized users.

## INTRODUCTION

Clinical drug research with children balances between the advancement of knowledge – and consequently possible improvement in clinical care – and the protection of a vulnerable population. On the one hand children are relatively incapable of protecting their own interest and therefore need to be protected from harm and exploitation in research.[14] On the other hand, clinical drug research is essential to generate sufficient evidence for improvements in pediatric care and drug dosing. Current estimates of off-label or unlicensed use of drugs range between 10 % and 60 % in the pediatric population.[24] Precisely because clinical drug research with children is a precarious enterprise, special attention needs to be given to the informed consent process.

Informed consent is one of the ethical cornerstones of human research. It represents the ethical principle of respect for persons: persons are treated as autonomous agents and persons with diminished autonomy are protected.[46] In the case of research with children, this means that their parents (or legal guardians) have to consent for them. This does not mean that children should be excluded from or ignored in the informed consent process. The United Nations Convention on the Rights of the Child states that children who are capable of forming their views have a right to express those views in any proceedings affecting the child directly.[48] Since they are the ones undergoing the research burden and risk, constructions of co-consent and assent are introduced in ethical and legal legislation to do justice to the opinion of children [1, 49, 56].

The process of informed consent and assent in clinical research with children might be clear in theory, in practice it is not. The question remains how to design this process of information and consent/assent as to include the perspective of children and their parents. Their perspective is vital, since they have the key role in decision-making on research participation. One way of taking their perspective into account is to look at the motivations children and their parents have to endorse or decline participation in pediatric clinical research. When professionals know to which aspects of research children and their parents attach importance, they know what information is relevant for their decision. And this knowledge may enable professionals involved in research to better tailor the process of recruitment and informed consent/assent to the perspective and needs of parents (or legal guardians) and children.

To our knowledge no comprehensive systematic review exists on these motivating and discouraging factors for children and their parents to decide to participate in clinical drug research. Two narrative reviews exist on why parents enroll their child in research.[16, 20] Both reviews show personal benefit and altruism as most important motivations of parents to enroll their child in research. However, these narrative reviews are not comprehensive nor systematically handled. Also, these reviews do not consider children’s motivations and are not focused on pharmacological research.

Therefore, we aimed to pool the existing empirical literature on motivations of children and their parents to consent or dissent to participation in clinical drug research. This systematic review attempts to answer the following research question: *What are motivating and discouraging factors for children and their parents to decide to participate in clinical drug research*?

## METHODS

This systematic review is reported in accordance with the Preferred Reporting Items for Systematic Review and Meta-analyses (PRISMA) statement [32]. The extra supplemental material provides the PRISMA checklist for this manuscript (online resource 1).

### Data sources and search strategy

We searched for peer-reviewed English-language articles using Embase, Medline, Web of Science, Pubmed, PsycINFO and the Cumulative Index to Nursing and Allied Health Literature (CINAHL) for empirical studies investigating the motivations (motivating and discouraging factors) of children and their parents to consent or dissent to participation in clinical drug trials. The search strategy was developed in collaboration with an information specialist of the Medical Library.

The search strategy was based on 3 concepts: 1) motivation for participation; 2) clinical drug research; 3) children and parents. The search strategy in Embase was as follows: (’refusal to participate’/de OR ’patient participation’/de OR ’parental consent’/de OR (((refus* OR decision* OR decid* OR allow* OR reason* OR motivat* OR willing* OR assent* OR consen* OR dissent* OR attitude* OR view* OR perspective* OR choos* OR choice*) NEAR/6 (participat* OR nonparticipat* OR enrol*))):ab,ti OR ((conflict/de OR ’motivation’/de OR drive/de OR ’informed consent’/de) AND (participat* OR nonparticipat* OR enrol*):ab,ti)) AND (’clinical trial (topic)’/exp OR ’pharmacological science’/exp OR ’clinical research’/de OR ((RCT* OR trial* OR scien* OR research*) NEAR/11 (participat* OR enrol*)):ab,ti OR ((’science in general’/de OR research/de OR ’medical research’/de OR ’human experiment’/de) AND (pharmacology/exp OR ’drug therapy’/exp OR (drug* OR pharmaco* OR medication* OR psychopharmacolog*):ab,ti))) AND (child/exp OR newborn/exp OR adolescent/exp OR adolescence/exp OR ’child behavior’/de OR ’child parent relation’/de OR (adolescen* OR infan* OR newborn* OR (new NEXT/1 born*) OR baby OR babies OR neonat* OR child* OR kid OR kids OR toddler* OR teen* OR boy* OR girl* OR minors OR underag* OR (under NEXT/1 ag*) OR juvenil* OR youth* OR kindergar* OR puber* OR pubescen* OR prepubescen* OR prepubert* OR pediatric* OR paediatric* OR school* OR preschool* OR highschool*):ab,ti). Searches in the other databases were based on the Embase search terms.

The extra supplemental material provides the exact search strategies in each database (online resource 2). We performed the initial search on March 20th 2013 and updated it on August 22th 2014.

### Inclusion and exclusion criteria

Studies were included when they addressed empirical data of: 1) children and/or parents on; 2) motivations for dissent or consent; 3) to participation in clinical drug research. We excluded articles with: 1) No empirical data; 2) Participation in only other clinical research than drug research; 3) Participation in vaccination studies (this religiously debated subject might confound results); 4) Narrative reviews.

### Study selection

**Fig. 1:**
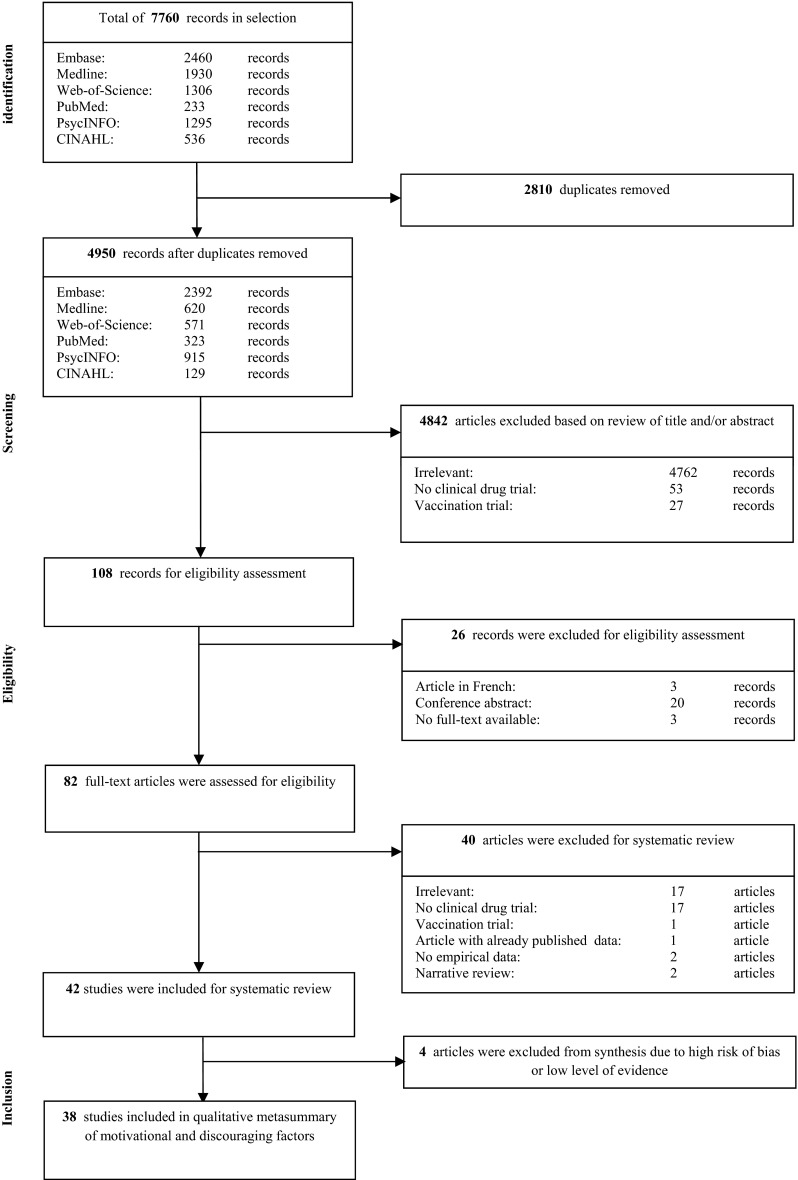
PRISMA flow diagram of study identification, screening, selection and inclusion

After identification of records from the search strategy, duplicates were removed from the retrieved records. In the screening phase, two reviewers (KT and WB) independently screened titles and abstracts of identified records for relevance to the research question. In case of discrepancy between the primary reviewers, a third reviewer (SvdV) decided upon inclusion for further eligibility assessment. In the eligibility phase, two reviewers (KT and WB) independently assessed full-text articles for eligibility. Again, in case of discrepancy between the primary reviewers, a third reviewer (SvdV) decided upon inclusion for systematic review. The PRISMA flow diagram presented in Fig. 1 shows the process of study selection: identification, screening, eligibility assessment and inclusion.

### Data extraction and study quality assessment

We extracted relevant data from the articles eligible for systematic review with the use of a data extraction form. A template of this form can be found in the extra supplemental material (online resource 3). The main outcome measures extracted were motivating factors and discouraging factors mentioned by children and/or their parents (or legal guardians). Study population, in- and exclusion criteria, patient characteristics, study design, and other outcome factors besides motivating and discouraging factors were also extracted. We graded the level of evidence of individual studies according to levels set by the Dutch Institute for Healthcare Improvement (CBO) (as indicated in Tables 1 and 2) and critically appraised the eligible articles to determine study quality and risk of bias (according to the Critical Appraisal Skills Programme (CASP) checklists) [15]. Studies with a very low level of evidence (level “D” for quantitative studies or level “-”for qualitative studies) or high risk of bias (based on CASP checklists) were excluded from data-synthesis of motivating and discouraging factors.

**Table 1:** Level of evidence of quantitative studies

Level of evidence ^a^	Characteristics
A1	Systematic reviews involving at least two studies at A2 level, of which the results of separate studies are consistent
A2	Randomized comparative clinical studies of good quality (randomized, double-blind controlled trails) of sufficient size and consistency
B	Randomized clinical trials of mediocre quality, of insufficient size, or other comparative studies
C	Non-comparative studies
D	Expert opinion

^a^ levels according to those set by the Dutch Institute for Healthcare Improvement (CBO)

**Table 2:** Level of evidence of qualitative studies

Level of evidence ^a^	Characteristics
++	Credible meta-synthesis of qualitative studies
+	Credible study
+/-	Study of which credibility is dubious
-	Study of which credibility is minimal

^a^ levels according to those set by the Dutch Institute for Healthcare Improvement (CBO)

### Data-synthesis

We performed a qualitative metasummary to give an overview of the motivating and discouraging factors mentioned by children and their parents. A qualitative metasummary is a quantitatively oriented aggregation approach to research synthesis of descriptive findings from both quantitative and qualitative studies. [43] This approach of data-synthesis entails treating research reports as indexes of the studies conducted, and the research findings in these reports as indexes of the experiences of the persons who participated in those studies. Therefore this approach functions well for our research question concerning motivations for participation, answered by qualitative and quantitative research. First, we extracted motivations mentioned by children or their parents from the result sections of the eligible studies regardless of how many participants endorsed the reason. Second, we created draft lists of all mentioned motivations in all studies for motivating factors and discouraging factors. Third, we grouped these motivations per theme and presented them as aggregated data. These themes of motivating and discouraging factors were not predefined, but defined by the total of extracted data.

## RESULTS

### Study selection

Our initial search produced 4950 titles after removing duplicates. After title and abstract screening, 108 records remained for full-text eligibility assessment. After full-text review, 42 articles could be included for data-extraction and systematic review [2–13, 18, 21–23, 25–28, 30, 31, 33–36, 38–42, 44, 45, 47, 50–53, 55, 57, 58]. Fig. 1 shows in a PRISMA flow diagram the results of study identification, screening, eligibility assessment and inclusion. Extracted data from these 42 studies, including study characteristics, motivating and discouraging factors, level of evidence and critical appraisal, can be found in the evidence tables as extra supplemental material (online resource 4).

### Characteristics of included studies for systematic review

Of the 42 articles that were included for systematic review, 26 were quantitative studies (including 15 written questionnaires, 7 verbally administered questionnaires and 4 studies analyzing registries of consent/dissent) and 16 were qualitative studies (including 10 interview studies, 2 focus group studies, 1 interview and focus group study and 3 studies with secondary analysis of interviews (of which one is a case study)). The number of research subjects involved per study ranged from 1 to 81 in the qualitative studies, and from 20 to 448 in the quantitative studies. In 37 studies parents (or caregivers/or legal guardians) were questioned about their motivations compared to 16 studies in which children themselves were questioned. The age of the children questioned ranged between 6 and 21 years. The majority of these studies included children up to 18 years of age. Three studies included children up to 21 years of age [8, 31, 40] Although, in Europe, we do not consider these respondents children, these studies were included because the majority of the respondents in these 3 studies were below 18 years of age. Two studies did not define the age of their respondents [5, 36]). The included studies were very diverse with regard to research population and setting (e.g., PICU/NICU setting, patients with airway diseases, with diabetes mellitus). Studies concerning oncology patients were most prevalent. Parents and children who consented to research were questioned in 39 studies, while 24 studies questioned respondents who dissented to research participation. Some studies questioned respondents about drug research in general or on a hypothetical drug study protocol (vignettes). But the majority questioned respondents in daily practice about participation in a specific drug study protocol. Most studies entailed participation in drug protocols with a prospect of direct benefit for the participant, only 5 drug protocols were considered to have no prospect of direct benefit for the participants. Table 3 shows an overview of study characteristics. The extra supplemental material provides evidence tables including these 42 studies with extracted data (online resource 4).

**Table 3:** Study characteristics of 42 included studies for systematic review

	Characteristic	No. of studies	Studies
Type of study	*Quantitative study*	*26*	*See categories below*
	- Written questionnaires	15	Barakat, 2013; Berg, 2010; Buscariollo, 2012; Cain, 2005; Cherill, 2010; Hoberman, 2013; Read, 2009; Sammons, 2007; Tait, 1998; Tait, 2003; Truong, 2011; Van Stuijvenberg, 1998; Vanhelst, 2013; Wagner, 2006; Zupancic, 1997
	- Verbally administered questionnaires	7	Baren, 1999; Brody, 2005; Brody, 2012; Harth, 1999; Miller, 2013; Rothmier, 2003; Wendler, 2012
	- Secondary analysis of data	4	Menon, 2012; Norris, 2010; Peden, 2000; Wynn, 2010
	*Qualitative study*	*16*	*See categories below*
	- Interviews	10	Barrera, 2005; Broome, 2003; Cartwright, 2011; Koelch, 2009; Liaschenko, 2001; MacNeill, 2013; Masiye, 2008; Patterson, 2014; Pletsch, 2001; Pletsch, 2001 (2); Woodgate, 2010
	- Focus groups ^a^	3	Caldwell, 2003; Lebensburger, 2013
	- Secondary analysis of data ^b^	3	Deatrick, 2002; Hoehn, 2005; Oppenheim, 2005
Study population	Only parents/caregivers	26	Baren, 1999; Buscariollo, 2012; Cartwright, 2011; Caldwell, 2003; Deatrick, 2002; Harth, 1999; Hoehn, 2005; Lebensburger, 2013; Liaschenko, 2001; MacNeill, 2013; Masiye, 2008; Menon, 2012; Oppenheim, 2005; Pletsch, 2001 (2); Pletsch, 2001; Rothmier, 2003; Sammons, 2007; Tait, 1998; Truong, 2011; Tait, 2003; Van Stuijvenberg, 1998; Vanhelst, 2013; Woodgate, 2010; Zupancic, 1997; Wynn, 2010; Hoberman, 2013
	Only children	5	Broome, 2003; Cain, 2005; Cherill, 2010; Koelch, 2009; Miller, 2013
	Both	11	Barakat, 2013; Barrera, 2005; Berg, 2010; Brody, 2005; Brody, 2012; Norris, 2010; Patterson, 2014; Peden, 2000; Read, 2009; Wagner, 2006; Wendler, 2012
Setting	Oncology	11	Barrera, 2005; Berg, 2010; Broome, 2003; Deatrick, 2002; Liaschenko, 2001; Miller, 2013; Oppenheim, 2005; Pletsch, 2001; Read, 2009; Truong, 2011; Woodgate, 2010
	Diabetes mellitus	5	Broome, 2003; Buscariollo, 2012; Cain, 2005; Pletsch, 2001 (2); Pletsch, 2001
	Airway diseases	7	Barakat, 2013; Brody, 2005; Brody, 2012; Harth, 1999; MacNeill, 2013; Rothmier, 2003; Sammons, 2007
	Sickle cell disease	4	Barakat, 2013; Lebensburger, 2013; Patterson, 2014; Wynn, 2010
	PICU / NICU	4	Cartwright, 2011; Hoehn, 2005; Menon, 2012; Zupancic, 1997
	Sick and healthy children (not specified)	4	Caldwell, 2003; Cherill, 2010; Vanhelst, 2013; Wendler, 2012
	Anesthetics	3	Peden, 2000; Tait, 1998; Tait, 2003
	Emergency department	2	Baren, 1999; Van Stuijvenberg, 1998;
	Psychopharmacology	2	Koelch, 2009; Wagner, 2006
	Other ^c^	3	Masiye, 2008; Norris, 2010; Hoberman, 2013;
Type of drug research	Real life drug study protocol	33	Barrera, 2005; Berg, 2010; Broome, 2003; Cain, 2005; Cartwright, 2011; Deatrick, 2002; MacNeill, 2013; Harth, 1999; Hoberman, 2013; Hoehn, 2005; Koelch, 2009; Liaschenko, 2001; Masiye, 2008; Menon, 2012; Miller, 2013; Norris, 2010; Oppenheim, 2005; Peden, 2000; Pletsch, 2001; Pletsch, 2001 (2); Read, 2009; Rothmier, 2003; Sammons, 2007; Tait, 1998; Tait, 2003; Truong, 2011; Van Stuijvenberg, 1998; Vanhelst, 2013; Wagner, 2006; Wendler, 2012; Woodgate, 2010; Wynn, 2010; Zupancic, 1997
	Drug research in general	4	Sammons, 2007; Tait, 1998; Tait, 2003; Van Stuijvenberg, 1998
	Hypothetical drug study protocol	5	Baren, 1999; Brody, 2005; Brody, 2012; Lebensburger, 2013; Patterson, 2014
Prospect of direct benefit	Only studies with prospect of direct benefit	22	Baren, 1999; Brody, 2012; Cain, 2005; Cartwright, 2011; Harth, 1999; Hoberman, 2013; Hoehn, 2005; Koelch, 2009; MacNeill, 2013; Masiye, 2008; Norris, 2010; Patterson, 2014; Peden, 2000; Pletsch, 2001 (2); Rothmier, 2003; Sammons, 2007; Tait, 1998; Tait, 2003; Van Stuijvenberg, 1998; Wagner, 2006; Wynn, 2010; Zupancic, 1997
	Only studies with no prospect of direct benefit	5	Barrera, 2005; Berg, 2010; Deatrick, 2002; Miller, 2013; Oppenheim, 2005
	Both	7	Broome, 2003; Liaschenko, 2001; Menon, 2012; Pletsch, 2001; Truong, 2011; Vanhelst, 2013; Wendler, 2012
	Not specified	8	Barakat, 2013; Brody, 2005; Buscariollo, 2012; Caldwell, 2003; Cherill, 2010; Lebensburger, 2013; Read, 2009; Woodgate, 2010
Consenters or non consenters	Only non consenters	3	Peden, 2000; Norris, 2010; Menon, 2012
	Only consenters	18	Broome, 2003; Cain, 2005; Cartwright, 2011; Deatrick, 2002; Liaschenko, 2001; Masiye, 2008; Miller, 2013; Oppenheim, 2005; Pletsch, 2001; Pletsch, 2001 (2); Rothmier, 2003; MacNeill, 2013; Truong, 2011; Van Stuijvenberg, 1998; Vanhelst, 2013; Wagner, 2006; Wendler, 2012; Woodgate, 2010;
	Both	21	Barakat, 2013; Baren, 1999; Barrera, 2005; Berg, 2010; Brody, 2005; Brody, 2012; Buscariollo, 2012; Caldwell, 2003; Cherill, 2010; Harth, 1999; Hoberman, 2013; Hoehn, 2005; Koelch, 2009; Lebensburger, 2013; Read, 2009; Sammons, 2007; Tait, 1998; Tait, 2003; Patterson, 2014; Wynn, 2010; Zupancic, 1997

PICU Pediatric Intensive Care Unit; NICU Neonatal Intensive Care Unit

^a^ Study of Caldwell included also personal interviews; ^b^ Study of Oppenheim is a case study; ^c^ anorexia nervosa, malaria, vesico-ureteral reflux

### Study quality and risk of bias

The evidence tables in the extra supplemental material show level of evidence (based on classification in Tables 1 and 2) and critical appraisal (including risk of bias) for individual studies. Four studies were of insufficient quality and were excluded from the qualitative metasummary due to very low level of evidence (level ‘D” or “-”) and high risk of bias. We excluded one qualitative study because the credibility was minimal (level of evidence “-”): the presented data did not answer their research question and essential parts of the data were not presented (population consisted of patients with diabetes mellitus and cancer, but data from cancer patients were missing in the article). [8] We excluded three quantitative studies due to high risk of bias: no separate analysis of adult research subjects and children [5]; represented data did not support article conclusions [3]; and inclusion of a very specific study population (patients with Anorexia Nervosa) in which treatment and research motivations cannot be looked at separately [33]. After these four exclusions due to insufficient quality 38 studies remained for data synthesis (qualitative metasummary) of motivating and discouraging factors. [2–4, 6, 7, 9–12, 18, 21–23, 25–31, 34–36, 38–42, 44, 45, 47, 50–53, 55, 57, 58].

### Qualitative metasummary of motivating factors

Of the 38 articles eligible for qualitative metasummary 33 studies included motivating factors mentioned by parents to endorse research participation of their child. Ten studies included motivating factors mentioned by children themselves. The extracted motivating factors mentioned by parents and children in the individual studies can be found in the evidence table in the supplemental information. Tables 4 and 5 give an overview of the motivating factors for parents and children. Individual health benefit, altruism (including helping others and contributing to science), a general trust in research and the relation to researchers are mentioned by parents in the highest number of studies. Other common motivating factors mentioned by parents to endorse research participation of their child include: more contact with the medical team, benefit for parents themselves, a sense of minimal burden for their child, the opportunity of financial reimbursement, feelings of having no other option, and influence of family and friends. For children themselves the most frequently mentioned factor favoring research participation include personal health benefit, altruism and increasing comfort by participation. Other motivating factors mentioned in multiple studies by children are the relation to the researcher, influence of family and friends, a financial reimbursement, increasing their knowledge about their disease and a sense of curiosity. In one study children also mentioned the feeling of having no other option available.

**Table 4:** Metasummary of motivating factors mentioned by parents for participation of their child in clinical drug research

Motivating factor	No. of studies (total = 33)	Individual studies
Personal health benefit for child ^a^	31	Barakat, 2013; Baren, 1999; Barrera, 2005; Brody, 2005; Brody, 2012; Buscariollo, 2012; Caldwell, 2003; Cartwright, 2011; Deatrick, 2002; Harth, 1999; Hoberman, 2013; Hoehn, 2005; Lebensburger, 2013; Liaschenko, 2001; MacNeill, 2013; Masiye, 2008; Oppenheim, 2005; Patterson, 2014; Pletsch, 2001; Pletsch, 2001 (2); Read, 2009; Sammons, 2007; Tait, 1998; Tait, 2003; Truong, 2011; Van Stuijvenberg, 1998; Vanhelst, 2013; Wagner, 2006; Woodgate, 2010; Wynn, 2010; Zupancic, 1997
Altruism ^b^	26	Baren, 1999; Barrera, 2005; Buscariollo, 2012; Caldwell, 2003; Cartwright, 2011; Deatrick, 2002; Harth, 1999; Hoberman, 2013; Hoehn, 2005; Liaschenko, 2001; MacNeill, 2013; Patterson, 2014; Pletsch, 2001; Pletsch, 2001 (2); Read, 2009; Rothmier, 2003; Sammons, 2007; Tait, 1998; Tait, 2003; Truong, 2011; Van Stuijvenberg, 1998; Vanhelst, 2013; Wendler, 2012; Woodgate, 2010; Wynn, 2010; Zupancic, 1997
Trust in safety of research	12	Barakat, 2013; Buscariollo, 2012; Cartwright, 2011; Harth, 1999; Hoberman, 2013; Hoehn, 2005; MacNeill, 2013; Patterson, 2014; Tait, 1998; Truong, 2011; Vanhelst, 2013; Zupancic, 1997
Relation to researcher	12	Buscariollo, 2012; Caldwell, 2003; Harth, 1999; Hoberman, 2013; Masiye, 2008; Read, 2009; Sammons, 2007; Tait, 1998; Tait, 2003; Truong, 2011; Woodgate, 2010; Van Stuijvenberg, 1998
More contact with medical team	8	Buscariollo, 2012; Caldwell, 2003; Harth, 1999; Lebensburger, 2013; MacNeill, 2013; Masiye, 2008; Wynn, 2010; Woodgate, 2010
Benefit for parents themselves	5	Harth, 1999; Oppenheim, 2005; Rothmier, 2003; Wagner, 2006; Van Stuijvenberg, 1998;
Minimal burden for child	4	Patterson, 2014; Pletsch, 2001 (2); Read, 2009; Woodgate, 2010
Financial reimbursement	5	Brody, 2012; Buscariollo, 2012; Harth, 1999; Masiye, 2008; Wagner, 2006
Felt as only option ^c^	4	Cartwright, 2011; Deatrick, 2002; Liaschenko, 2001; Oppenheim, 2005
Influence of family and friends	3	Buscariollo, 2012; Harth, 1999; Read, 2009

^a^ factor mentioned in studies with and without prospect of direct benefit; ^b^ In 3 studies specifically defined as no motivating factor; ^c^ all studies were in oncology setting

**Table 5:** Metasummary of motivating factors mentioned by children for participation in clinical drug research

Motivating factor	No. of studies (total = 10)	Individual studies
Personal health benefit^a^	8	Barrera, 2005; Brody, 2005; Brody, 2012; Cain, 2005; Miller, 2013; Patterson, 2014; Read, 2009; Wagner, 2006
Altruism	6	Cain, 2005; Miller, 2013; Patterson, 2014; Read, 2009; Wagner, 2006; Wendler, 2012
Increasing comfort	4	Cain, 2005; Koelch, 2009; Miller, 2013; Read, 2009
Relation to researcher	3	Miller, 2013; Read, 2009; Wagner, 2006
Influence of family and friends	3	Cain, 2005; Read, 2009; Wagner, 2006
Financial reimbursement	3	Brody, 2005; Brody, 2012; Wagner, 2006
Increasing knowledge	2	Cain, 2005; Wagner, 2006
Curiosity	2	Cain, 2005; Koelch, 2009
Felt as only option	1	Miller, 2013

^a^ factor mentioned in studies with and without prospect of direct benefit

### Qualitative metasummary of discouraging factors

Of the 38 articles eligible for qualitative metasummary 24 studies included discouraging factors mentioned by parents for research participation of their child. Six studies included discouraging factors mentioned by children themselves. These include motivations mentioned by respondents who dissented to research participation, but also discouraging factors mentioned by respondents who did participate but considered these factors as negatively influencing their decision. The extracted discouraging factors mentioned by parents and children in the individual studies can be found in the evidence table in the extra supplemental material. Tables 6 and 7 give an overview of the discouraging factors for parents and children. Fear of potential risks, a general distrust in research, logistical aspects and disruption of daily life and fear of burden for their child are mentioned by parents in the highest number of studies. Other common discouraging factors mentioned in multiple studies by parents for research participation of their child include: decision considered to be too stressful, a fear of randomization, no prospect of direct benefit for their child, financial constraints and a discomfort with being a proxy. Discouraging factors incidentally mentioned by parents are for example a discord between guardians, religious constraints or privacy issues. For children themselves the most frequently mentioned factors discouraging research participation include fear of burden for themselves and disruption of their daily life, feeling like a “guinea pig” and a fear of risks. Other discouraging factors incidentally mentioned by children are the prospect of no direct benefit, no understanding of the study, preference for one arm and the decision considered to be too stressful.

**Table 6:** Metasummary of discouraging factors mentioned by parents for participation of their child in clinical drug research

Discouraging factor	No. of studies (total = 24)	Individual studies
Fear of risks	14	Baren, 1999; Brody, 2005; Brody, 2012; Buscariollo, 2012; Caldwell, 2003; Harth, 1999; Hoehn, 2005; Lebensburger, 2013; MacNeill, 2013; Patterson, 2014; Pletsch, 2001 (2); Read, 2009; Tait, 1998; Tait, 2003;
Distrust in research (“guinea pig”)	11	Baren, 1999; Caldwell, 2003; Harth, 1999; Hoehn, 2005; Lebensburger, 2013; Menon, 2012; Peden, 2000; Read, 2009; Sammons, 2007; Tait, 1998; Wynn, 2010
Logistics / disruption of daily life ^a^	11	Baren, 1999; Brody, 2005; Caldwell, 2003; Patterson, 2014; Harth, 1999; Lebensburger, 2013; Peden, 2000; Pletsch, 2001; Read, 2009; Tait, 1998; Wynn, 2010
Burden for child	9	Barrera, 2005; Brody, 2005; Buscariollo, 2012; Menon, 2012; Oppenheim, 2005; Peden, 2000; Pletsch, 2001 (2); Read, 2009; Woodgate, 2010
Decision too stressful	7	Hoberman, 2013; Lebensburger, 2013; Menon, 2012; Pletsch, 2001; Read, 2009; Sammons, 2007; Tait, 1998
Fear of randomization	6	Caldwell, 2003; Lebensburger, 2013; MacNeill, 2013; Sammons, 2007; Tait, 1998; Wynn, 2010
No direct benefit for child ^b^	5	Baren, 1999; Barrera, 2005; MacNeill, 2013; Read, 2009; Wynn, 2010
Financial constraints	5	Baren, 1999; Buscariollo, 2012; Harth, 1999; Tait, 1998; Wynn, 2010
Discomfort with proxy consent	2	Buscariollo, 2012; Caldwell, 2003

^a^ for child *and* rest of family; ^b^ of which 3 are defined as studies with no prospect of direct benefit

**Table 7:** Metasummary of discouraging factors mentioned by children for participation in clinical drug research

Discouraging factor	No. of studies (total = 6)	Individual studies
Burden / disruption of daily life	4	Brody, 2005; Koelch, 2009; Read, 2009; Patterson, 2014
Feeling like a “guinea pig”	3	Koelch, 2009; Peden, 2000; Read, 2009
Fear of risks	3	Brody, 2005; Brody, 2012; Patterson, 2014
Decision too stressful	1	Read, 2009
No understanding	1	Read, 2009
No direct benefit	1	Read, 2009
Preference for one arm	1	Peden, 2000

## DISCUSSION

This systematic review shows that the most frequently mentioned motivating factors for parents to endorse their child’s participation in clinical drug research are: health benefit for their child, altruism, a trust in research, and their relation to the researcher. Most frequently mentioned motivating factors for children to participate are: personal health benefit, altruism and increasing comfort. Fear of risks, a distrust in research, logistical aspects and disruption of daily life are mentioned most frequently as discouraging factors to endorse participation of their child by parents. Burden and disruption of daily life, feeling like a “guinea pig” and fear of risks were most frequently mentioned as discouraging factors by children.

One of the most important ethical criteria on which a research ethics board (REB) should evaluate a research protocol, is whether the objective outweighs the risk and burden to the research subjects: called a consideration of proportionality. In other words: an REB assesses the predictable risk and burden to the research subjects in comparison to the foreseeable benefit to them and to other individuals or groups affected by the investigated condition.[56] Our review shows that this proportionality is also considered by parents and children in their own individual decision about research participation; personal health benefit and altruism are the most frequently mentioned motivating factors and risk and burden are frequently mentioned as discouraging factors. In 7 studies the weighing of these factors (proportionality) is even specifically mentioned by parents.[2, 11, 27, 35, 38, 39, 45] In 2 studies children mention explicit this proportionate weighing.[25, 35].

### Burden of participation

The results also show that it is not only burden for the *participating child* that influences the decision, but also burden for *parents themselves and the rest of their family*. Professionals involved in pediatric research need to be aware that when a child participates in research, a lot of the burden falls on the shoulders of parents: e.g., they need to be present at the hospital, they are often the ones filling in the diaries. This burden may negatively affect the decision of parents to let their child participate in research. That is also true for logistical aspects and disruption of the lives of the whole family. Parents are the ones absent from work and they need to make sure that other family members are looked after when their child participates in research. Parents mention for example “*the inconveniences of trial participation”* [11] or “*too many visits*” [57] as reasons for dissent.

### General trust and mistrust in research

Issues of general trust in research or general mistrust (often explained with wordings as “guinea pig”) influence the decision of parents and children greatly. These issues of trust and mistrust might indicate that their decision is not a weighing of factors but an a priori decision. This idea of an a priori decision was also suggested a few years ago by W. Pinxten in his thesis. [37] The general trust of children and their parents in research needs to be protected by careful evaluation of study protocols by an REB beforehand. A proper evaluation system beforehand ensures that the studies offered to parents and children are of such quality that their trust in research is well-founded.

### Personal health benefit

Personal health benefit is one of the most important motivators for parents and children themselves to participate in clinical drug research. This is of course not problematic when the study has therapeutic objectives, but is problematic when no prospect of direct benefit exists.

In all 3 studies with no prospect of direct benefit (all oncology phase 1 studies) where parents were questioned, possible health benefit for their child was a motivating factor. [4, 18, 34] In the study of Deatrick et al. most parents saw their child’s participation in the trial as *“a means of providing treatment to prolong life, though an uncertain treatment”*. [18] In the study of Barrera et al. families main motivator for enrolling in phase 1 trials was *“hope for a cure or prolongation of the child’s life and their belief that participating would ensure continuity of care”*. [4] Since the objective of these phase 1 studies is safety assessment and not effectiveness, and because of the fading boundary between research and care, therapeutic misconception is a clear danger in these studies.[17] Adequate information on the rationale of the study is therefore essential. Professionals involved in clinical research need to be aware that the line between hope and reality is thin. As illustrated by an interviewed mother from the study from Oppenheim: *“…the study was proposed as an alternative, and we accepted it to avoid the operation and to gain more time, even a week, but not really believing that it could cure F”*. [34].

Children are also vulnerable to therapeutic misconception, as shown in two studies with no prospect of direct benefit in which children themselves mention therapeutic benefit as an important motivating factor. [4, 31].

### Altruism

Helping others or contributing to science is an important motivation for parents to endorse participation in clinical drug research. However, 3 studies concluded that altruism was explicitly not a factor in the decision of parents. [2, 34, 52] Altruistic motivations might be overestimated in this review. These could be socially desirable answers. Remarkable is the finding of Truong et al., that parents with a child in a phase 3 study mention altruistic motivations more often than parents with a child in phase 1 studies. [47].

Helping others and contributing to science were also mentioned frequently by children as a motivation for participation in research. For example, more than 80 % of the questioned children in the study of Wendler et al. indicated that finding better treatments for others was important to their decision to enroll. [53] Two studies that showed altruistic motivations in children questioned children starting at an age of 6 years old. This might indicate that children can be altruistic at a much younger age than currently suggested.[54] Unfortunately, the studies addressing altruism had very wide age ranges (6-18 years) and no stratified analyses for age groups. It would be interesting to look deeper into the role of altruistic motivations of children in pediatric research.

### Relation to researcher

Parents and children mention their relation to the researcher quite often as a factor influencing their decision to participate. This should not be a problem if they ask him/her for advice or feel safe with him/her. But it is problematic when parents and children use words as *“I felt pressure*”[40] or even *“They told me to”*[52]. This means that parents and children may feel less free when asked to participate. The effect of this relationship on their decision needs to be considered even more carefully when the roles of researcher and treating physician converge in one, which is often the case in pediatrics. [19].

### More contact with medical team

Parents mentioned quite often more contact with the medical team as a favoring factor for endorsing research participation . For example, in the study by Masiye et al, some participants felt that if they would refuse to participate in the study, their child might not receive attention from the healthcare workers whenever they would visit the hospital again. [29] And some parents in Caldwell’s stated that their child would be better monitored when he/she would be in the trial.[11] This suggests that parents think their child is better looked after or treated when in research. Parents need to be aware that (non) participation in research does not affect their regular treatment. In our opinion, a patient should not be dependent on research to get the attention he/she wishes for in a treatment setting.

### Felt as only option

Striking is the observation that parents sometimes endorse participation because it feels for them as if they do not have an option. [12, 18, 27, 31, 40] This can be a problematic factor, when there are other options available of which parents are not sufficiently aware of. But in certain hospital settings (for example oncology setting) participation in research is indeed the only option parents and children have opposed to palliative care. Furthermore, some children and parents can only accept the child’s upcoming death when they have tried all available options. One parent illustrated this clearly in a study by Deatrick et. al.: “*There wasn’t really a choice in my mind because if I choose to not do anything then I would have been choosing to let her go and I’m not ready for that.”*[18].

### No direct benefit for child

Surprisingly, this review shows parents can refuse participation because they do not expect benefit for their child. It is striking that this is mentioned in 3 randomized phase 3 studies (where a prospect of benefit exists). [3, 28, 57] A possible explanation could be that parents have a preference for the experimental intervention arm (compared to standard or placebo arm) and are suspicious of the randomization since it does not guarantee them access to the experimental intervention arm. This is illustrated in the study by Baren et. al in which parents mention fear of receiving less than optimal treatment in the study as a discouraging factor for participation [3].

### Limitations and strengths of this review

This systematic review gives a comprehensive overview of motivating and discouraging factors for children and their parents to consent to clinical drug research. Since we aimed to give an overview of all the available empirical literature on this topic, there is a large variety in drug trials, settings and populations of the studies. This heterogeneity in studies might complicate the interpretation of the pooled data, but we feel it is essential to pool these heterogeneous results, since it reflects the diverse practice of pediatric drug trials.

Because of challenges in the search strings, we limited our research question to participation in pharmacological research. Therefore it is uncertain whether we can extrapolate these results to other medical research (including observational research and other interventional research).

We reported in the qualitative metasummary the number of studies citing a specific factor. The number of articles reporting a specific factor may not represent the importance of this factor to the research participants. Besides that, given the wide range in the number of research participants per study, an increasing number of studies citing a factor, does not necessarily reflect more parents or children mentioning this factor. However, qualitative metasummary is still the best way to pool this kind of data from qualitative and quantitative empirical studies. To get more insight in the motivations of parents and children qualitative research is of essential value and a large portion of the data in this review comes from qualitative data. Therefore, this way of pooling the data does justice to the diversity in qualitative and quantitative research available for answering our research question. By including qualitative and quantitative research the strengths of both types of research are combined; in depth results and possibility of unanticipated motivations from qualitative research, and large sample sizes and standardization from quantitative research.

## Conclusion

It is essential that professionals during the recruitment and informed consent/assent process pay attention to the motivating and discouraging factors children and their parents have for participation in clinical drug research When professionals know more about the motivations of parents and children to endorse or decline participation in clinical drug research, professionals know which aspects of research parents and children attach importance to and what information is of relevance for their decision. This information can then be used by professionals in the informed consent materials and conversations. When children and their parents are being informed about the aspects of research to which they attach importance, they may reach a decision more consistent with their own values. Therefore, the attention to these motivating and discouraging factors makes the *informed consent/assent* of parents and children more *informed*, which thus increases the *moral* value of informed consent/assent.

Besides leading to an increase in the *moral v*alue of informed consent, paying attention to the motivations of children and their parents for participation in clinical drug research can also be of instrumental value. By adapting the study protocol, the recruitment and the informed consent process to the needs and wishes of children and their parents, participation rates will probably increase (and dropout rates can decrease). For example, by diminishing logistical barriers (which this review shows, are mentioned often by parents as negatively influencing their decision) at the setup of the study, parents and children will probably be more inclined to participate. Therefore, adapting the research protocol, recruitment and informed consent process to the needs of children and their parents may lead to *more* informed consents.

This systematic review gives a comprehensive overview of the available empirical data on motivating and discouraging factors for parents and children to consent/assent to clinical drug research. But it also shows us that specific populations are underrepresented in this field of research. Further research is needed in diverse populations and research fields (for example healthy children, children with chronic disease such as cystic fibrosis, and critically ill children). This future research should specifically focus on the factors that shape the decision of children themselves, since research with children on this topic is scarce. Although children cannot consent by themselves, they can assent and we shouldn’t forget to listen to them. They are the ones bearing the burden and risk during participation in clinical drug research, and possible beneficiaries of the research.

## Electronic supplementary material

Below is the link to the electronic supplementary material.

ESM 1 (PDF 80.6 kb)

ESM 2 (PDF 38.8 kb)

ESM 3 (PDF 22.4 kb)

ESM 4 (PDF 176 kb)

## ABBREVIATIONS

CASP: Critical Appraisal Skills Programme
CBO: Dutch Institute for Healthcare Improvement
CINAHL: Cumulative Index to Nursing and Allied Health Literature (CINAHL)
NICU: Neonatal Intensive Care Unit
No.: Number
PICU: Pediatric Intensive Care Unit
PRISMA: Preferred Reporting Items for Systematic Review and Meta-analyses
REB: Research Ethics Board
