# Supplementary material for: Motivations of children and their parents to participate in drug research: a systematic review
Source: Eur J Pediatr. 2016 Apr 4;175:599–612. doi: 10.1007/s00431-016-2715-9 (PMC4839044; doi:10.1007/s00431-016-2715-9)
Supplement: Supplementary file 2 — (PDF 38.8 kb) [file 431_2016_2715_MOESM2_ESM.pdf]

Extra supplemental material 2

Search strings per database

Database / Search engine	Search string
**Embase**	('refusal to participate'/de OR 'patient participation'/de OR 'parental consent'/de OR (((refus* OR decision* OR decid* OR allow* OR reason* OR motivat* OR willing* OR assent* OR consen* OR dissent* OR attitude* OR view* OR perspective* OR choos* OR choice*) NEAR/6 (participat* OR nonparticipat* OR enrol*))):ab,ti OR ((conflict/de OR 'motivation'/de OR drive/de OR 'informed consent'/de) AND (participat* OR nonparticipat* OR enrol*):ab,ti)) AND ('clinical trial (topic)'/exp OR 'pharmacological science'/exp OR 'clinical research'/de OR ((RCT* OR trial* OR scien* OR research*) NEAR/11 (participat* OR enrol*)):ab,ti OR (('science in general'/de OR research/de OR 'medical research'/de OR 'human experiment'/de) AND (pharmacology/exp OR 'drug therapy'/exp OR (drug* OR pharmaco* OR medication* OR psychopharmacolog*):ab,ti))) AND (child/exp OR newborn/exp OR adolescent/exp OR adolescence/exp OR 'child behavior'/de OR 'child parent relation'/de OR (adolescen* OR infan* OR newborn* OR (new NEXT/1 born*) OR baby OR babies OR neonat* OR child* OR kid OR kids OR toddler* OR teen* OR boy* OR girl* OR minors OR underag* OR (under NEXT/1 ag*) OR juvenil* OR youth* OR kindergar* OR puber* OR pubescen* OR prepubescen* OR prepubert* OR pediatric* OR paediatric* OR school* OR preschool* OR highschool*):ab,ti)
**Medline**	("refusal to participate"/ OR "patient participation"/ OR "parental consent"/ OR (((refus* OR decision* OR decid* OR allow* OR reason* OR motivat* OR willing* OR assent* OR consen* OR dissent* OR attitude* OR view* OR perspective* OR choos* OR choice*) ADJ6 (participat* OR nonparticipat* OR enrol*))).ab,ti. OR (("Conflict (Psychology)"/ OR "motivation"/ OR "drive"/ OR "Intention"/ OR exp "informed consent"/) AND (participat* OR nonparticipat* OR enrol*).ab,ti.)) AND (exp "clinical Trials as Topic "/ OR "Biomedical Research"/ OR ((RCT* OR trial* OR scien* OR research*) ADJ11 (participat* OR enrol*)).ab,ti. OR (("Science"/ OR research/ OR exp "Human Experimentation"/) AND (exp pharmacology/ OR pharmacology.xs. OR exp "drug therapy"/ OR drug therapy.xs. OR (drug* OR pharmaco* OR medication* OR psychopharmacolog*).ab,ti.))) AND (exp child/ OR exp infant/ OR adolescent/ OR exp "child behavior"/ OR exp "Parent-Child Relations"/ OR (adolescen* OR infan* OR newborn* OR (new ADJ born*) OR baby OR babies OR neonat* OR child* OR kid OR kids OR toddler* OR teen* OR boy* OR girl* OR minors OR underag* OR (under ADJ ag*) OR juvenil* OR youth* OR kindergar* OR puber* OR pubescen* OR prepubescen* OR prepubert* OR pediatric* OR paediatric* OR school* OR preschool* OR highschool*).ab,ti.)
**Web-of-Science**	TS=(((((refus* OR decision* OR decid* OR allow* OR reason* OR motivat* OR willing* OR assent* OR consen* OR dissent* OR attitude* OR view* OR perspective* OR choos* OR choice*) NEAR/6 (participat* OR nonparticipat* OR enrol*)))) AND (((RCT* OR trial* OR scien* OR research*) NEAR/11 (participat* OR enrol*))) AND ((adolescen* OR infan* OR newborn* OR new born* OR baby OR babies OR neonat* OR child* OR kid OR kids OR toddler* OR teen* OR boy* OR girl* OR minors OR underag* OR under age* OR juvenil* OR youth* OR kindergar* OR puber* OR pubescen* OR prepubescen* OR prepubert* OR pediatric* OR paediatric* OR school* OR preschool* OR highschool*)))
**PubMed**	(refus*[tiab] OR decision*[tiab] OR decid*[tiab] OR allow*[tiab] OR reason*[tiab] OR motivat*[tiab] OR willing*[tiab] OR assent*[tiab] OR consen*[tiab] OR dissent*[tiab] OR attitude*[tiab] OR view*[tiab] OR perspective*[tiab] OR choos*[tiab] OR choice*[tiab]) AND (participat*[tiab] OR nonparticipat*[tiab] OR enrol*[tiab]) AND (scien*[tiab] OR research*[tiab]) AND (adolescen*[tiab] OR infan*[tiab] OR newborn*[tiab] OR new born*[tiab] OR baby[tiab] OR babies[tiab] OR neonat*[tiab] OR child*[tiab] OR kid[tiab] OR kids[tiab] OR toddler*[tiab] OR teen*[tiab] OR boy*[tiab] OR girl*[tiab] OR minors[tiab] OR underag*[tiab] OR under age*[tiab] OR juvenil*[tiab] OR youth*[tiab] OR kindergar*[tiab] OR puber*[tiab] OR pubescen*[tiab] OR prepubescen*[tiab] OR prepubert*[tiab] OR pediatric*[tiab] OR paediatric*[tiab] OR school*[tiab] OR preschool*[tiab] OR highschool*[tiab]) AND publisher[sb]
**PsycINFO**	("Participation"/ OR "client participation"/ OR (((refus* OR decision* OR decid* OR allow* OR reason* OR motivat* OR willing* OR assent* OR consen* OR dissent* OR attitude* OR view* OR perspective* OR choos* OR choice*) ADJ6 (participat* OR nonparticipat* OR enrol*))).ab,ti. OR ((exp "Conflict"/ OR exp "motivation"/ OR "Intention"/ OR exp "informed consent"/) AND (participat* OR nonparticipat* OR enrol*).ab,ti.)) AND ("clinical Trials"/ OR ((RCT*

	OR trial* OR scien* OR research*) ADJ11 (participat* OR enrol*)).ab,ti. OR (("Sciences"/ OR Experimentation/ OR) AND (exp pharmacology/ OR exp "drug therapy"/ OR (drug* OR pharmaco* OR medication* OR psychopharmacolog*).ab,ti.))) AND (100.ag. OR 200.ag. OR "Child Psychology"/ OR exp "Parent-Child Relations"/ OR (adolescen* OR infan* OR newborn* OR new ADJ born* OR baby OR babies OR neonat* OR child* OR kid OR kids OR toddler* OR teen* OR boy* OR girl* OR minors OR underag* OR under ADJ ag* OR juvenil* OR youth* OR kindergar* OR puber* OR pubescen* OR prepubescen* OR prepubert* OR pediatric* OR paediatric* OR school* OR preschool* OR highschool*).ab,ti.)
**CINAHL**	(MH "refusal to participate"+ OR MH "Consumer Participation"+ OR (((refus* OR decision* OR decid* OR allow* OR reason* OR motivat* OR willing* OR assent* OR consen* OR dissent* OR attitude* OR view* OR perspective* OR choos* OR choice*) N6 (participat* OR nonparticipat* OR enrol*))) OR ((MH "Conflict (Psychology)"+ OR MH "motivation" OR MH "drive" OR MH "Intention" OR MH "consent"+) AND (participat* OR nonparticipat* OR enrol*))) AND (MH "clinical Trials"+ OR ((RCT* OR trial* OR scien* OR research*) N11 (participat* OR enrol*)) OR ((MH "Science" OR MH research) AND (MH "Pharmacy and Pharmacology"+ OR MH "drug therapy"+ OR (drug* OR pharmaco* OR medication* OR psychopharmacolog*)))) AND (MH child+ OR MH "child behavior"+ OR (adolescen* OR infan* OR newborn* OR (new N1 born) OR baby OR babies OR neonat* OR child* OR kid OR kids OR toddler* OR teen* OR boy* OR girl* OR minors OR underag* OR (under N1 age) OR juvenil* OR youth* OR kindergar* OR puber* OR pubescen* OR prepubescen* OR prepubert* OR pediatric* OR paediatric* OR school* OR preschool* OR highschool*))
