# Supplementary material for: Motivations of children and their parents to participate in drug research: a systematic review
Source: Eur J Pediatr. 2016 Apr 4;175:599–612. doi: 10.1007/s00431-016-2715-9 (PMC4839044; doi:10.1007/s00431-016-2715-9)
Supplement: Supplementary file 3 — (PDF 22.4 kb) [file 431_2016_2715_MOESM3_ESM.pdf]

Extra supplemental material 3

<u>Data extraction form</u>

Relevant aspect	Study No.:	
**Author and year**		
**Type of study**	☐ Qualitative study: …<br>☐ Quantitative study: …	
**Setting (description)**		
- Moment of questioning related to decision and participation		
- real life / hypothetical research / research in general		
- therapeutic / non therapeutic		
- parents and /or children		
- separate analysis of parents and children?		
- consenters / non consenters		
**Study for which participation is asked**		
**Study population**		
**Number of participants**		
**Inclusion criteria**		
**Exclusion criteria**		
**Patient characteristics**		
**Objective/ hypothesis**		
**Methods**		
**Motivating factors**	Parents:	Children:
**Discouraging factors**	Parents:	Children:
**Other outcome measures**		
**Possible confounders**		
**Critical appraisal (including risk of bias)** [a]		
**Level of evidence** [b]	Quantitative study:<br>☐ A<br>☐ B<br>☐ C<br>☐ D	Qualitative study:<br>☐ + +<br>☐ +<br>☐ + / -<br>☐ -

[a] With use of the Critical Appraisal Skills Programme (CASP) checklists [11];
[b] Levels according those set by the Dutch Institute for Healthcare Improvement (CBO)
