# Supplementary material for: Motivations of children and their parents to participate in drug research: a systematic review
Source: Eur J Pediatr. 2016 Apr 4;175:599–612. doi: 10.1007/s00431-016-2715-9 (PMC4839044; doi:10.1007/s00431-016-2715-9)
Supplement: Supplementary file 4 — (PDF 176 kb) [file 431_2016_2715_MOESM4_ESM.pdf]

Extra supplemental material 4

Evidence Tables

**Author, year**	**Methods**	**Outcomes**
**Barakat, 2013**	Study population: 103 children and 76 AYA's with Asthma or SCD and their 224 caregivers with and without prior research experience.<br>Inclusion criteria: ability to speak and read English.<br>Exclusion criteria: not mentioned.<br>Characteristics: consenting and non-consenting children (8-18 years) and parents.<br>Design: quantitative study; written questionnaires during regularly scheduled follow-up visits in clinic about research in general (including drug trials). Exploratory factor analysis to identify latent structures.	Motivating factors: patient benefit, trust in safety of research, the opportunity costs to engaging in research (parents).<br>Discouraging factors: mistrust of research and researchers (parents).<br>Other outcomes: proportionality, prior research exposure.<br>Confounding: not mentioned.<br>Level of evidence: B<br>Critical appraisal: large sample size, adapted questionnaire for children. No open ended questions, only opinion (yes/no) asked about statements. No descriptive results of questionnaire published, only the factors in the model.

Author, year	Methods	Outcomes
**Baren, 1999**	Study population: 227 parents of children being seen for minor traumatic injuries in 3 paediatric emergency departments.<br>Inclusion criteria: not mentioned.<br>Exclusion criteria: parents whose children were aged 16 years or older, sustained injuries raising suspicion of abuse, required IC admission or operative intervention.<br>Characteristics: consenting and non-consenting parents (mean age: 34 years).<br>Design: quantitative study; verbal questionnaires about participation in hypothetical clinical drug trial (RCT with Phenytoin).	Motivating factors: benefit to child (85%); benefit to other children (72%); further medical knowledge (60%).<br>Discouraging factors: fear of adverse effects (54%); don't want child to be a research subject (39%); need to discuss with family first (27%); can't decide unless in actual situation (26%); fear of less than optimal treatment(10%); opposition to medical research (9%); do not understand study (9%); religious beliefs 3 (4%); do not have time to participate 2 (3%); financial concerns (3%); language barrier (3%); prior bad experience with research (1%); prior bad experience with medical profession (1%); other (21%).<br>Other outcomes: ethnicity and household income associated with consent decision.<br>Confounding: hypothetical protocols.<br>Level of evidence: B<br>Critical appraisal: large population size; good thing that questioning of reasons was not predefined. hypothetical study, and critical ill children were excluded, therefore maybe not applicable to real situation.
**Barrera, 2005**	Study population: 9 families of children with recurrent disease in hematology/oncology unit eligible for phase 1 oncology trial.<br>Inclusion criteria: not mentioned.<br>Exclusion criteria: families of children identified by staff as being too ill or too overwhelmed and families of children under age of 7.<br>Characteristics: 8 families consenting, 1 family non-consenting; 7 mothers, 2 fathers (mean age: 34 years); 3 children (7-15 years).<br>Design: qualitative study; Individual, semi structured interviews after decision of participation. Identification of themes in interviews.	Motivating factors: parents: hope for a cure, continuing care, focus on quality of life; children: hope for a cure.<br>Discouraging factors: focus on quality of life (parents).<br>Other outcomes: ambivalence/uncertainty of parents.<br>Confounding: hypothetical decision, could be different in real consent situations (due to extreme psychological and emotional distress).<br>Level of evidence: +<br>Critical appraisal: small number of interviewed people, especially children. No extensive description of results, only 4 major themes mentioned.

Author, year	Methods	Outcomes
**Berg, 2010**	Study population: 53 subjects who participate in a phase 1 anticancer drug study.<br>Inclusion criteria: consent or dissent to PK sampling.<br>Exclusion criteria: not mentioned.<br>Characteristics: 8 adult subjects, 4 adolescents and 38 parents/legally authorized representative; consenting and non-consenting.<br>Design: quantitative study; written questionnaire administered within 4 weeks after consent to phase 1 drug study about (non)consenting to extra PK sampling within study.	Motivating factors: 97% defined altruistic reasons as very or extremely important:; 83% ranked “no extra pain or harm to child” as very or extremely important.<br>Discouraging factors: Large percentage defined time and need for an extra IV as important concern.<br>Other outcomes: additional comments by subjects.<br>Confounding: no attempt to control for demographic factors.<br>Level of evidence: C<br>Critical appraisal: bad quality; no distinction between children, parents and adult participants; content of questionnaire not clear.
**Brody, 2005**	Study population: 36 adolescent-parent dyads (predominantly mothers) of which children had a prior diagnosis of asthma.<br>Inclusion criteria: child with prior diagnosis of asthma.<br>Exclusion criteria: not mentioned.<br>Characteristics: 2 guardians, 34 parents (30-60 years) and 36 adolescents (11-17 years); consenters and non-consenters.<br>Design: quantitative study; separate interviews about willingness to participate after presentation of 9 hypothetical asthma research protocols.	Motivating factors: parents: perception of research benefit (45%), Children: perception of research benefit (40%), financial compensation (10%).<br>Discouraging factors: parents: concern over hassle (25%), risk (25%), discomfort (3%); children: concern over hassle (35%), risk (10%), discomfort (7%).<br>Other outcomes: 60% of the time parents and adolescents held concordant views on participation decisions.<br>Confounding: parents and children were interviewed separately, this differs from actual process; order of protocols was systematically varied, but could have an influence on decision.<br>Level of evidence: C<br>Critical appraisal: positive and negative responses of willingness to participate are grouped together.

**Author, year**	**Methods**	**Outcomes**
**Brody, 2012**	Study population: 111 adolescents with asthma and their 111 parents.<br>Inclusion criteria: prior diagnosis of asthma, English speaking , child between 11 and 17 years of age.<br>Exclusion criteria: not mentioned.<br>Characteristics: mean age adolescents 13.6 (range:10-17); parents mean age 41.9 years, 93% at least high school diploma; consenters and non-consenters.<br>Design: quantitative study; development of conceptual model of research participation decisions is developed . adolescents and parents are interviewed about hypothetical asthma research protocol (informed by video).	Motivating factors: benefit and financial compensation are factors in model for adolescents and parents.<br>Discouraging factors: perceived risks is factor in model for adolescents and parents.<br>Other outcomes: 67% of parents and adolescents agreed on the participation decision.<br>Confounding: demographic variables, level of comprehension.<br>Level of evidence: C<br>Critical appraisal: small sample size to build a model on with that many variables; single hypothetical protocol.
**Broome, 2003**	Study population: 34 children and adolescents with DM or hematological malignancies requiring treatment who are/were previous enrolled in research.<br>Inclusion criteria: consent from parent, > 7 years of age, diagnosed with a health condition requiring treatment, enrolled in a research study within the last 2 months, speaks English, at least one English-speaking parent who is also willing to be interviewed.<br>Exclusion criteria: not mentioned.<br>Characteristics: age range: 8-22 years; 23 with hematologic malignancy,10 with DM; only consenters.<br>Design: qualitative study; tape-recorded semi structured interviews at home or in hospital about various drug studies.	Motivating factors: the monetary incentive that was offered (DM patients).<br>Discouraging factors: time involved and number of needle sticks (DM patients).<br>Other outcomes: influence/relationship with parents.<br>Confounding: not mentioned.<br>Level of evidence: -<br>Critical appraisal: bad quality, only results from DM patients presented, limited information from interviews, article does not answer their research question.

Author, year	Methods	Outcomes
**Buscariollo, 2012**	Study population: 166 parents of children with DM1.<br>Inclusion criteria: not mentioned.<br>Exclusion criteria: not mentioned.<br>Characteristics: 81% female, 90% Caucasian; consenters and non-consenters;<br>Design: quantitative study; 48-item written questionnaire including open-ended, yes/no and 5-point responses to assess parental attitudes towards DM1 clinical trials and willingness to participate (research in general and hypothetical trials).	Motivating factors: potential benefit for their own child (92%), potential benefit for other children in the future (87%), opportunity to contribute to science (43%), influences of family and friends (31%), financial compensation (32%), increased physician access at no additional cost (47%).<br>Discouraging factors: risk of side effects associated with trial participation (57%), discomfort with consent by proxy or making decisions about trial participation for their children (27%), fear of having to pay for research treatment (30%), lack or cost of transportation (30%), child's fear of receiving injections (19%).<br>Other outcomes: prediction factors for WTP; comfort scores with different types of trials.<br>Confounding: possible non-response bias effects.<br>Level of evidence: B<br>Critical appraisal: extensive description of results, but very low response rate.
**Cain, 2005**	Study population: 36 children who had participated in a trial comparing insulin detemir with NPH in a multi-injection therapy for type 1 diabetes.<br>Inclusion criteria: from UK and Ireland; age between 6-17 years.<br>Exclusion criteria: not mentioned.<br>Characteristics: consenting children; 6-11 years: 17%; 12-14 years: 58%; 15-17 years: 25%.<br>Design: quantitative study; non-validated, 23-item postal questionnaire, child friendly written with graded scales, numerical scales and free text responses to examine attitudes and experiences to drug trial participation.	Motivating factors: "I wanted to improve my blood sugar control": 30%; "I thought it would be interesting": 21%; "I wanted to help other people with diabetes": 19%; "My mum/dad thought it would be a good idea": 9%; "I wanted to know more about my diabetes": 6%; "My friend was doing it": 2%; "I wanted to use the pen": 4%; "I wanted to be helpful in any way I could": 2%; "I wanted more flexibility with my insulin/diabetes": 6%.<br>Discouraging factors: not mentioned.<br>Other outcomes: 81% would take part in a future trial; experiences during participation, information provided.<br>Confounding: trial participants are a self-selecting group and sample used in this study is small; therefore may not be representative of the general paediatric population<br>Level of evidence: C<br>Critical appraisal: child friendly questionnaire used, only consenters questioned, high response rate; non-validated questionnaire.

Author, year	Methods	Outcomes
**Caldwell, 2003**	Study population: 33 parents with sick children from children's hospital and with healthy children from local primary school.<br>Inclusion criteria: not mentioned.<br>Exclusion criteria: not mentioned.<br>Characteristics: healthy children: 27%, acute illness: 18%, chronic illness: 15%, cancer: 18%, RCT participants: 21%; 73% with previous research experience.<br>Design: qualitative study; 4 focus groups and 5 individual interviews to explore attitudes towards child's participation in RCT's; data coded using constant comparative methods and further examined to identify emergent overarching themes.	Motivating factors: perceived benefits, doctor factors, child factors.<br>Discouraging factors: perceived risks, trial factors, parental factors.<br>Other outcomes: proportionality.<br>Confounding: not mentioned.<br>Level of evidence: +<br>Critical appraisal: comprehensive description of results; paid attention to different backgrounds and settings; no distinction between focus groups and individual interviews and no distinction based on previous research experience.
**Cartwright, 2011**	Study population: 16 parents of 12 infants born with complications who had participated in an RCT (immunotherapy, ventilation, hypothermia).<br>Inclusion criteria: parents read and speak English fluently; parents' infants had participated in a RCT in the previous 18 months while receiving intensive care in the NICU.<br>Exclusion criteria: not mentioned.<br>Characteristics: 10 mothers (27-36 years), 6 fathers (27-36 years); all white Europeans, all consenters.<br>Design: qualitative study; semi-structured face-to-face interviews after trial participation; open-ended and closed questions.	Motivating factors: themes from interviews.<br>Discouraging factors: not mentioned.<br>Other outcomes: immediate reactions, interaction with clinician, implications of RCT, effect of RCT.<br>Confounding: parental responses may have been affected by time lag between participation and interview.<br>Level of evidence: +<br>Critical appraisal: small sample size, elaborate results from interviews, no discouraging factors mentioned.
**Cherill, 2010**	Study population: 98 healthy children at secondary school and 117 children with a chronic illness at outpatient clinic or hospital.<br>Inclusion criteria: child and parent in agreement to participate.<br>Exclusion criteria: not mentioned.<br>Characteristics: healthy children: median age 13 (11-16) years. Chronic ill children: median age: 14 (11-16) years.<br>Design: quantitative study; written questionnaire about viewpoints of research in general (including drug trial) including closed questions and 3 hypothetical scenarios.	Motivating factors: Helping others was the most common reason given for taking part in clinical trials. Altruistic nature of children in both groups was similar.<br>Discouraging factors: not mentioned.<br>Other outcomes: Alarming: 57-63% of children would participate in a cancer drug trials as a healthy volunteer.<br>Confounding: not mentioned.<br>Level of evidence: B<br>Critical appraisal: bad quality, only small part of results published; abstract and discussion mention altruistic motives, but not results not presented.

Author, year	Methods	Outcomes
**Deatrick, 2002**	Study population: 21 parents of children participating in phase 1 oncology trial.<br>Inclusion criteria: not mentioned.<br>Exclusion criteria: not mentioned.<br>Characteristics: 19 mothers, 2 fathers; children: 2-18 years. Only consenters.<br>Design: qualitative study; descriptive cross-sectional study with secondary analysis techniques to analyze existing qualitative data from two studies of parents decision making at end of life for their children with cancer.	Motivating factors: prolong life for their child / delaying death; buying time for another therapy; providing treatment; working a miracle; desire to help other children with cancer in the future; practical concerns (including location and proximity of available treatment, ability to secure treatment in the near future and issues related to quality of life), child's physical condition (good shape).<br>Discouraging factors: child's physical condition (weak).<br>Other outcomes: all parents saw limited choices or no choices in the decisions about whether to enter their child in a phase 1 clinical trial.<br>Confounding: not mentioned.<br>Level of evidence: +<br>Critical appraisal: article only mentions some aspects of parents views; no systematic representation; but a lot of examples from interviews.
**Harth, 1999**	Study population: 68 parents who had volunteered their child for a randomized, double, blind, placebo controlled trial of ketotifen (new drug for asthma) and 42 parents who had refused this participation.<br>Inclusion criteria: not mentioned.<br>Exclusion criteria: not mentioned.<br>Characteristics: majority Caucasian, majority between (20-29 years of age).<br>Design: quantitative study; verbal questionnaire consisting of 48 structured and 2 open ended sections to assess perceptions, attitudes, and health seeking behavior of the parents.	Motivating factors: to benefit my own child: N=61; dissatisfaction with current treatment: N=56; to learn more about medical treatment: N=51; liked the people conducting the trial: N=49; to meet people: N=45; trust in the hospital: N=33; to gain better access to health care: N=26; advice of family doctor: N=10; advice of others: N=8<br>reimbursement of travel cost: N=8.<br>Discouraging factors: fear of side effects of the new drug: N=40; inconvenience of frequent visits: N=35; dislike of becoming involved: N=33; lack of time: N=23; distrust of modern medicine: N=22; loss of privacy: N=14; Not interested: N=10; distrust of the hospital: N=8; extra cost entailed: N=5.<br>Other outcomes: difference between consenters and non-consenters: socio-demographic characteristics, health seeking behavior, availability of social support.<br>Confounding: no selection bias in recruitment.<br>Level of evidence: B<br>Critical appraisal: moment of questionnaire in relation to decision not clear. Large response rate, no response bias expected.

Author, year	Methods	Outcomes
**Hoberman, 2013**	Study population: 120 parents who were asked to provide consent for their child's participation in a randomized controlled trial of antimicrobial prophylaxis for vesicoureteral reflux.<br>Inclusion criteria: not mentioned.<br>Exclusion criteria: not mentioned.<br>Characteristics: 48 consenters, median age: 31 years; 62 non-consenters, median age 33 years; majority Caucasian.<br>Design: quantitative study; written questionnaire consisting of Likert scales and VAS. Examining difference between consenters and non-consenters in 7 constructs governing the decision to provide consent.	Motivating factors: significant differences between consenters and non-consenters:<br>trust in research; perceiving researcher as friendly/professional; benefit to their child; benefit to others (altruism); importance of study.<br>Discouraging factors: significant differences between consenters and non-consenters: interference of study with standard of care; feelings of anxiety and decisional uncertainty.<br>Other outcomes: child-, parent- and study characteristics, parental perception of the study, parental understanding of study design, external influences, decision making process.<br>Confounding: overrepresentation of higher levels of education in non-consenters; less than 50% response rate (no difference between consenters/non-consenters.<br>Level of evidence: B<br>Critical appraisal: good quality. Questionnaire based on previous research. But very low response rate and no in and exclusion criteria mentioned.
**Hoehn, 2005**	Study population: 34 parents of 24 neonates having cardiothoracic surgery invited to participate in a study evaluating the impact of prenatal diagnosis on parental permission for neonatal cardiac surgery.<br>Inclusion criteria: not mentioned.<br>Exclusion criteria: not mentioned.<br>Characteristics: 14 fathers, 20 mothers; majority Caucasian.<br>Design: qualitative study; Qualitative analysis of the unsolicited comments (spontaneously mentioned) of parents regarding reasons for agreeing or declining to participate in research studies.	Motivating factors: societal benefit (N=18/53%) (pro reason); individual benefit to their infant (N=16/47%) (pro reason); perception of no risk of harm (N=9/26%) (neutral reason).<br>Discouraging factors: risk of study participation (N=10/29%) (con reason); Anti-experimentation (feeling like a guinea pig) (N=4/12%) (con reason).<br>Other outcomes: comparison of reasons for consenters and non-consenters.<br>Confounding: not mentioned.<br>Level of evidence: +<br>Critical appraisal: strong point: spontaneous comments, no predefined reasons. No linking of reasons to specific studies. Very little recall bias.

Author, year	Methods	Outcomes
**Koelch, 2009**	Study population: 19 child-parent dyads enrolled in an RCT with investigational drug or an open-label trial with licensed drug (psychopharmacology)<br>Inclusion criteria: not mentioned.<br>Exclusion criteria: not mentioned.<br>Characteristics: children's mean age: 11 years, range: 7-15 years; all boys; 15 consenters, 3 non consenters, 1 undecided.<br>Design: qualitative study; interviews by use of MacArthur Competence Assessment Tool for Clinical Research; analyzed with qualitative content analysis.	Motivating factors: hopes for improvement of their own behavior based on experience (with benefit for themselves and/or for their families); Comfort (new medication easier to handle); explorative behavior/sensation seeking (the chance to test something new).<br>Discouraging factors: changes in treatment settings; Time spent; Burden of study examinations (blood-drawings); feeling like a guinea pig.<br>Other outcomes: proportionality, understanding, appreciation.<br>Confounding: IQ and experience influences reasoning.<br>Level of evidence: +<br>Critical appraisal: comprehensive elaboration of interview results. Children and parents interviewed, but results of reasoning of parents not described, only reasons of children.
**Lebensburger, 2013**	Study population: 14 parents or guardians of children (with SCD) with no prior experience with clinical trials or hydroxyurea therapy<br>Inclusion criteria: not mentioned<br>Exclusion criteria: not mentioned<br>Characteristics: 3 male, 11 female; average age: 42 years (31-56); all African-American.<br>Design: qualitative study; 3 focus groups addressing 7 main questions and a mock recruitment pamphlet for a hypothetical feasibility trial of hydroxyurea for prevention of secondary silent cerebral infarcts.	Motivating factors: improvement child's life, discuss trial with other participants, increased clinic visits<br>Discouraging factors: General mistrust of research studies, emotional issues (burden for child), practical issues (time required, missing work etc.), randomization, long term unknown risks,<br>Other outcomes: -<br>Confounding: possibly response bias.<br>Level of evidence: +<br>Critical appraisal: Weak point: no in- and exclusion criteria and little info on patient characteristics. Strong point: accurate and visible coding of themes.

Author, year	Methods	Outcomes
**Liaschenko, 2001**	Study population: 12 fathers of children diagnosed with cancer and involved in a clinical cancer research study at a children's hospital.<br>Inclusion criteria: fathers with a child who: was diagnosed with cancer, had participated in clinical research within last year, was at least 8 years of age, had at least one parent who was legally authorized to give informed consent.<br>Exclusion criteria: not mentioned.<br>Characteristics: majority Caucasian, children's mean age: 13.5 years. All consenters.<br>Design: qualitative study; focused interviews in private setting to explore meanings of research and reasons for participation.	Motivating factors: altruism; no other option available; Possibility of and hope for direct improvement without significantly increasing the risk of more harm, Maximize the child's chance of survival.<br>Discouraging factors: not mentioned.<br>Other outcomes: description of life context, description of meanings of research<br>Confounding: reasons for participation interact with meanings of participation and type of research.<br>Level of evidence: +<br>Critical appraisal: well defined methodology; Only brief description of results from interviews, very aggregated.
**MacNeill, 2013**	Study population: 42 parents of children participating in a randomized double blind placebo-controlled trial of montelukast for preschool wheeze<br>Inclusion criteria: not mentioned.<br>Exclusion criteria: not mentioned.<br>Characteristics: 10 males, 32 females; mean age: 36 years; 20 Bangladeshi, 10 white UK, 12 other.<br>Design: qualitative study; semi-structured interviews to compare the motives and experiences of different ethnic groups.	Motivating factors: Benefit to child (21/42). Benefit to others (15/42); trust in the research team (3/42); Route to additional information, treatment and attention.<br>Discouraging factors: No benefit, adverse effects, randomization to placebo.<br>Other outcomes: experience of consent process; understanding research process, consulting others. Difference between ethnic groups.<br>Confounding: No non-consenters and Bangladeshi parents underrepresented.<br>Level of evidence: +<br>Critical appraisal: Good quality; transparent: coding example in article. Elaborate description of results.

**Author, year**	**Methods**	**Outcomes**
**Masiye, 2008**	Study population: 81 female guardians of children participating in the Intermittent Prevention Therapy post-discharge (IPTpd) Malaria Research<br>Inclusion criteria: not mentioned.<br>Exclusion criteria: not mentioned.<br>Characteristics: 39 from rural area, 42 urban area; mean age rural: 29 years, mean age urban: 28 years; education rural: 6 years, education urban: 9 years; All concenters.<br>Design: qualitative study; 8 focus groups to assess the reasons why mothers enroll their children in malaria clinical research and how family members or relatives are involved in the decision making process.	Motivating factors: majority wanted their children to receive better treatment, participants wanted to benefit from the material and monetary incentives that were given, sense of trust in the health workers, attention by health care workers<br>Discouraging factors: Not mentioned<br>Other outcomes: perspective on the informed consent process and role of partner in decision making process.<br>Confounding: not mentioned<br>Level of evidence: +<br>Critical appraisal: sufficient quality; Weak point: no in- and exclusion criteria mentioned . Strong point: inclusion of themes and quotations of participants.
**Menon, 2012**	Study population: 54 non-consenting legal guardians who were approached for consent for any ongoing PICU research.<br>Inclusion criteria: not mentioned.<br>Exclusion criteria: surveys and chart audits.<br>Characteristics: 54 non-consenters; Children's age: 0.6 years.<br>Design: Quantitative study; prospective, observational study with recording of demographic data and unsolicited reasons stated by legal guardians for consent refusal.	Motivating factors: not mentioned.<br>Discouraging factors: Guardian too stressed: N=24; Blood taking required for study: N=13; Medication administration required for study: N=3; Radiation required for study: N=2; Guardian does not agree with research: N=8; Already in another study: N=6<br>Discord between guardians: N=2; Child has been through enough: N=7<br>Other: N=28.<br>Other outcomes: description of patient and study demographics.<br>Confounding: not mentioned.<br>Level of evidence: B<br>Critical appraisal: Positive: unsolicited reasons, no suggestions. Only reasons for refusal stated by non-consenters.

Author, year	Methods	Outcomes
**Miller, 2013**	Study population: 20 adolescents with cancer who were offered participation in a phase 1 trial.<br>Inclusion criteria: permission from parent and adolescent.<br>Exclusion criteria: not mentioned.<br>Characteristics: median age: 17.8 years; 7 participants: 14-17 years, 13 participants:18-21 years; majority male and Caucasian; all consenters.<br>Design: Quantitative study; verbal questionnaire with closed and open-ended questions to examine adolescents perspectives.	Motivating factors: Positive clinical effect: N=15 (75%); No other options: N=9 (45%); Positive impact on quality of life: N=8 (40%); Few or fewer side effects: N=8 (40%); Logistics related to participation (e.g., ‘‘It’s easy to do.’’): N=6 (30%); Previous testing/availability of trial drug: N=5 (25%); To help science and other children: N=4 (20%); Doctor’s recommendation: N=3 (15%); Other: N=5 (25%).<br>Discouraging factors: not mentioned.<br>Other outcomes: Experience of process, expectations.<br>Confounding: perceptions are likely not biased by trial participation or change in health status (due to little time between consent and interview).<br>Level of evidence: C<br>Critical appraisal: elaborate interpretation of results. Positive that reasons were not predefined, but an open question.
**Norris, 2010**	Study population: 20 adolescents and their parents refused to participate in an RCT involving olazapine for the adjunctive treatment of anorexia nervosa.<br>Inclusion criteria: not mentioned.<br>Exclusion criteria: not mentioned.<br>Characteristics: all female, median age 15.4 years; all non-consenters.<br>Design: Quantitative study; secondary descriptive analysis of reasons provided by patients and their parents for refusal of study participation. already available data.	Motivating factors: not applicable<br>Discouraging factors: Adolescents: Not interested in taking any psychotropic medication / fears associated with effects of medication (i.e. weight gain): N=7; Refused randomization N=2; Fears associated with participation in research trial N=2. Parents: Not interested in or wanting child on any psychotropic medication / fears associated with side effects of medication (i.e. potential for diabetes) N=7; Refused randomization N=2.<br>Other outcomes: 55% (n=11) of refusals were patient (adolescent) driven.<br>Confounding: not mentioned.<br>Level of evidence: C<br>Critical appraisal: Bad quality; little information, too broad description of reasons, small sample size, very specific population, with specific reasons for refusal (probably related to effect of trial (weight gain), not generalizable.

Author, year	Methods	Outcomes
**Oppenheim, 2005**	Study population: mother who accepted her daughter to be included in a phase 1-2 oncology trial.<br>Inclusion criteria: not applicable.<br>Exclusion criteria: not applicable.<br>Characteristics: mother of a child 7 years old treated since age of 2 for malignant germinal tumor, consented to trial.<br>Design: Qualitative study; secondary analysis of an interview of a mother with a psycho-oncologist to discuss relational, psychological en ethical issues of phase 1-2 trials.	Motivating factors: motivating themes identified in interview.<br>Discouraging factors: discouraging themes identified in interview.<br>Other outcomes: other themes.<br>Confounding: not mentioned.<br>Level of evidence: +<br>Critical appraisal: Only 1 subject, but elaborate analysis of interview.
**Patterson, 2014**	Study population: 23 caregivers of patients with SCD , 16 paediatric patients with SCD and (13 AYA's with SCD)<br>Inclusion criteria: fluent in English<br>Exclusion criteria: not mentioned<br>Characteristics: 21 male/2 female caregivers, median age: 42.1 years; 8 female/8 male children, median age: 12.6 years; majority African American. Consenters and non-consenters.<br>Design: Qualitative study; semi-structured interviews asking about previous research experience and reasons to enroll and assessment of 2 vignettes (placebo controlled drug trial and psychosocial study).	Motivating factors: parents consenting to drug vignette: potential benefit (42.9%), altruism (43.5%), trust (13.3%), manageable study demands; children consenting to drug vignette: potential benefit (37.5%), altruism (37.5%), manageable study demands.<br>Discouraging factors: parents dissenting to drug vignette: potential harm (71.9%), unmanageable study demands (28.1%); children dissenting to drug vignette: potential harm (55.6%), unmanageable study demands (44.4%).<br>Other outcomes: reasons for previous participation, ranking of statements. Weighing of proportionality.<br>Confounding: sampling bias. Results from hypothetical studies might not correlate with actual decision.<br>Level of evidence: +<br>Critical appraisal: Sufficient quality.; no actual responses of participants visible, only coding groups. But elaborate results presented.

Author, year	Methods	Outcomes
**Peden, 2000**	Study population: 448 children presenting for venipuncture to a day-care ward, who are approached to take part in a clinical trial evaluating a new local anesthetic agent and declined to take part in the trial.<br>Inclusion criteria: inclusion criteria for trial.<br>Exclusion criteria: not mentioned.<br>Characteristics: not mentioned. Only non-consenters.<br>Design: Quantitative study; analysis of records kept by research nurse of each parent and child who was approached and where consent was declined.	Motivating factors: not applicable<br>Discouraging factors: Parents: Time: N=157, Do not wish to be involved in the trial: N=74, Parents not wanting the child to have local anesthetic: N=31; Children: Child requesting local anesthetic cream previously used: N=70, Child unhappy to take part in trial: N=28, Child upset or shy: N=24.<br>Other outcomes: practical problems of non-inclusion.<br>Confounding: not mentioned.<br>Level of evidence: C<br>Critical appraisal: Sufficient quality; large population, but not a clear distinction between reasons of parents and children and practical problems of non-inclusion.
**Pletsch, 2001**	Study population: 33 mothers of children diagnosed with cancer or DM1 and involved in clinical research studies (including drug trials).<br>Inclusion criteria: not mentioned.<br>Exclusion criteria: not mentioned.<br>Characteristics: 24 mothers of child with cancer (child's mean age: 12.5 years), 9 mothers of child with DM1 (child's mean age: 10.6 years); all consenters.<br>Design: Qualitative study; Semi-structured interviews with mothers. Narrative analysis techniques used to identify patterns in experiences.	Motivating factors: Cancer group: to save the life of their child, benefit they were looking was life over death; DM1: consider personal benefits that might accrue for their child, as well as societal benefits, contribution to improved knowledge about diabetes care for other children.<br>Discouraging factors: DM1: some mothers thought that diabetes was all the burden a child should be asked to bear, inconveniences.<br>Other outcomes: other themes related to experiences, proportionality.<br>Confounding: not mentioned.<br>Level of evidence: +<br>Critical appraisal: Positive: open questions about reasons, not predefined. Elaborate comparison between the two groups; No info about in- and exclusion criteria. Number of participants not consistent in article.

Author, year	Methods	Outcomes
**Pletsch, 2001 (2)**	Study population: 9 mothers of children with DM1 and involved in clinical research (2 drug trials) at children's hospital.<br>Inclusion criteria: child at least 9 years of age and prior experience with participating in a clinical trial.<br>Exclusion criteria: not mentioned.<br>Characteristics: Mean age mothers: 42 years, all European and high school graduates; mean age children: 10.6 years (range: 9-13 years).<br>Design: Qualitative study; semi-structured interviews with mothers to identify patterns influencing consent to clinical research.	Motivating factors: Continued well-being of their child; must be some direct and immediate advantage for their child (personal benefit); opportunities.<br>Discouraging factors: Risks.<br>Other outcomes: 3 steps in decision making; interaction parent/child.<br>Confounding: sample cannot be taken as representative of the general population of mothers of chronically ill children nor all mothers of children with diabetes.<br>Level of evidence: +<br>Critical appraisal: Strength: 2 members independently performed analysis, very elaborate description and analysis of results; Weakness: very homogenous group.

Author, year	Methods	Outcomes
**Read, 2009**	Study population: 86 Adolescents and young adults diagnosed with cancer and 409 parents of children with cancer at 5 paediatric oncology centers.<br>Inclusion criteria: recall of being offered participation in health research; >12 years of age<br>Exclusion criteria: not mentioned.<br>Characteristics: AYA's median age: 18 (12-22) years (50% consenters); parents median age: 40 (15-74) years (64% consenters).<br>Design: Quantitative study; validated postal questionnaires to describe personal factors that may influence decision to participate. Descriptive statistics and associations between demographic characteristics and attitudes were described.	Motivating factors: I thought it would help others: AYA: 67%, P: 85%; I thought it would help me/my child: AYA: 26%, P: 60%; I thought it would not add too much discomfort: AYA: 19%, P: 20%; I felt pressure from my doctor to take part: AYA: 19%, P: 21%; I felt pressure from my family or friends to take part: AYA: 7%, P: 3%; I thought it would not add too much time: AYA: 6%, P: 13%; I did not have any choice taking part in the study: AYA: 2%, P: NA; Other: AYA: 1%, P: 8%.<br>Discouraging factors: Study required too much of my time: AYA: 45%, P: 13%; I had too much else to think about at the time: AYA: 36%, P: 21%; I did not think it would help me: AYA: 18%, P: 13%; Study required me to undergo increased discomfort: AYA: 18%, P: 26%; I did not want to be a guinea pig: AYA: 9%, P: 11%; Study too hard to understand: AYA: 9%, P: 5%; I did not trust the person offering me the study: AYA: 0%, P: 3%; Too risky: AYA: 0%, P: 13%; Other: AYA: 1%, P: 37%.<br>Other outcomes: factors influencing participation of parents themselves in research.<br>Confounding: altruistic motives could have been influenced by social acceptability.<br>Level of evidence: B<br>Critical appraisal: Large sample size. Very little response on discouraging factors. AYA's include minors and adults.
**Rothmier, 2003**	Study population: 44 parents or guardians of children less than 18 years of age who were currently involved in clinical asthma research.<br>Inclusion criteria: not mentioned.<br>Exclusion criteria: not mentioned.<br>Characteristics: parent's mean age: 40 years, majority Caucasian females; children's age between 4 and 7 years. All consenters<br>Design: Quantitative study; 2-page questionnaire administered in person containing 14 liker-type questions. Factors influencing parental consent were ranked on liker-scale.	Motivating factors: Most influential: Learn more about disease; Help medical knowledge; Newest drugs.<br>Discouraging factors: not mentioned.<br>Other outcomes: factors less convincing/ important influencing decision.<br>Confounding: not mentioned.<br>Level of evidence: C<br>Critical appraisal: Small sample size for quantitative study. No distinction made between negatively influencing and not influencing factors.

Author, year	Methods	Outcomes
**Sammons, 2007**	Study population: 136 parents of children who were recruited for a multicenter randomized equivalence trial comparing oral and intravenous treatment for pneumonia.<br>Inclusion criteria: children aged 6 months to 16 years with fever, respiratory symptoms or signs and radiologically confirmed pneumonia.<br>Exclusion criteria: not mentioned.<br>Characteristics: children's median age: 2.0 years (6 months-12 years). Consenters and non-consenters<br>Design: Quantitative study. Short postal questionnaire administered after trial participation, with free text questions and agree/disagree questions to assess what motivates parents to consent to an RCT.	Motivating factors: benefit to all children in the future: 32%; contribution to science: 27%; benefit to their own child: 19%; asked by a doctor: 13%; no reason not to: 7%.<br>Discouraging factors: wanting a specific treatment for their child / unwilling to undergo randomization (N=25); Do not want to participate in a trial (N=2); too distressed by their child's admission (N=2); PIF stated that the ethics committee would have access to their child's data (N=1).<br>Other outcomes: factors influencing decision in future studies.<br>Confounding: possible overestimation of positive attitudes, due to low response rate; recall bias (different recall windows).<br>Level of evidence: C<br>Critical appraisal: good quality of questions (mix of open-ended and closed questions).<br>Little information about study population.
**Tait, 2003**	Study population: 505 parents/guardians who had been approached to allow their child to participate in any one of 18 ongoing clinical anesthesia or surgery studies.<br>Inclusion criteria: not mentioned.<br>Exclusion criteria: not mentioned.<br>Characteristics: parent's mean age: 37.1 years; child's mean age: 7.2 years; 411 consenters, 94 non-consenters.<br>Design: Quantitative study; questionnaire filled in by parents during participation of their child in trial to identify factors influencing their decision.	Motivating factors: positive predictors for consent: perceived benefits to child; perceived importance of study.<br>Discouraging factors: negative predictor for consent: perceived risk of study.<br>Other outcomes: factors influencing decision for future studies; interaction parent/child.<br>Confounding: not mentioned.<br>Level of evidence: B<br>Critical appraisal: Large sample size, large amount of data collected, elaborate description of results.

Author, year	Methods	Outcomes
**Tait, 1998**	Study population: 246 parents/guardians who had been approached for permission to allow their child to participate in any one of a number of anesthesia research studies currently underway at the C.S. Mott Children's Hospital.<br>Inclusion criteria: not mentioned.<br>Exclusion criteria: not mentioned.<br>Characteristics: No demographic differences between consenters and non-consenters; 168 consenters, 78 non-consenters.<br>Design: Quantitative study; written questionnaire detailing reasons for their decision. Reasons were analyzed by principal component analysis.	Motivating factors: Minimal risk to child: 86.1%; Other children might benefit: 83.7 %; Study was explained well: 77.9%; Understood the study: 77.5%; Study was important: 67.9%; Contribute to medical science: 69.1%; Risk was small in relation to the importance of the study: 68.8%; Child might benefit: 51.2%; The researcher put you at ease: 44.7%; Sufficient time to decide: 36.1%; Child would receive "better" care: 13.0%; Felt uncomfortable saying "no": 4.4 %; Felt obligated to consent: 3.1%.<br>Discouraging factors: Fear for safety of child: 61,6%; Potential risk to child: 59,7%<br>Randomized to placebo or drug: 40,8%; Another "thing" to worry about: 35,6%; Fear of unknown: 35.2%; Study might interfere with care: 21,1%; Insufficient time to decide: 15,3%; Child would be a "guinea pig": 15,3%; Distrust of medical system: 5,6 %; Moral/religious reasons: 4,2 %; Did not understand study: 2,8%; No privacy to decide: 2,8%; No financial compensation: 1,4%; Researcher made you feel uncomfortable: 1,4%.<br>Other outcomes: factors influencing decision for future studies.<br>Confounding: not mentioned.<br>Level of evidence: B<br>Critical appraisal: large sample size and large response rate. Reliability of questionnaire tested.

Author, year	Methods	Outcomes
**Truong, 2011**	Study population: (205 adult patients) and 48 parents of paediatric cancer patients participating in phase I, II, or III clinical trials of cancer-directed therapy.<br>Inclusion criteria: consent to a qualified cancer trial within the previous 14 days.<br>Exclusion criteria: consent obtained by an investigator of the present study, consent obtained in another language than English, email-address outside USA, participant removed from trial within 14 days, participant died.<br>Characteristics: parent's mean age: 38.8 years, majority Caucasian and female; 20% phase I, 18% phase 2, 961% phase 3. All consenters.<br>Design: Quantitative study; postal questionnaire including 9 statements of motivations for participation (with a focus on altruism).	Motivating factors: To help future patients: 50%; To help advance medical science: 49%; To receive medical benefits: 48%; I trust the doctor: 46%; I trust this hospital: 54%<br>To maintain hope: 54%.<br>Discouraging factors: not mentioned.<br>Other outcomes: Being motivated primarily by altruism was positively correlated with phase of trial.<br>Confounding: limited socio-demographic diversity, therefore limiting generalizability.<br>Level of evidence: C<br>Critical appraisal: predefined reasons (socially acceptable answering?); Focus on altruism in results, therefore other reasons are underexposed.
**Van Stuijvenberg, 1998**	Study population: 181 parents or guardians who had volunteered their child for a randomized, double blind, placebo controlled trial of ibuprofen to prevent febrile seizure recurrences.<br>Inclusion criteria: children between 1 and 4 years old; with a recognized risk of febrile seizure recurrence; parents were Dutch or English speaking; child had visited the emergency room of the Sophia Children's Hospital in Rotterdam or the Juliana Children's Hospital in Den Haag because of a febrile seizure.<br>Exclusion criteria: not mentioned.<br>Characteristics: 181 mothers (median age: 32.6 years) and 155 fathers (median age: 35.6 years) of 181 children; majority West-European; all consenters.<br>Design: Quantitative study; postal questionnaire with structured and semi-structured questions to assess the quality of the informed consent process.	Motivating factors: Contribution to clinical science (n = 92; 51%); Benefit for their own child (n = 58; 32%); Give something in return for the care of their child (n = 12; 7%); Benefit for other children in future (n = 5; 3%); Benefit for the parent (n = 6; 3%); The doctor asked (n = 6; 3%); No major reason (n = 2; 1%).<br>Discouraging factors: not mentioned.<br>Other outcomes: comprehensibility of information, awareness of 6 major trial characteristics, perception of the informed consent procedure; factors influencing decision for future studies.<br>Confounding: possible overestimation of positive experiences, possibility of socially desirable answers.<br>Level of evidence: C<br>Critical appraisal: Good quality; sufficient sample size, questionnaire partially validated.

Author, year	Methods	Outcomes
**Vanhelst, 2013**	Study population: 261 parents of children who participated in paediatric clinical research at Lille Clinical Investigation Center of the Lille University Hospital.<br>Inclusion criteria: Paediatric clinical research study conducted between 2004 and 2007; Child aged between 1 and 18 years.<br>Exclusion criteria: Paediatric clinical research studies involving neonates hospitalized in the intensive care unit; Children enrolled in oncology paediatric clinical research studies, who were considered to be a highly specific group of patients with an immediate, potentially poor outcome; Babies enrolled in industrial milk formula studies; Other studies involving children aged less than one year.<br>Characteristics: 126 parents of healthy children, 99 ambulant sick children, 36 non-ambulant sick children. All consenters.<br>Design: Quantitative study; postal questionnaire with closed questions to identify motivating factors linked to child health status that affected consent to participation.	Motivating factors: Direct benefits to the parent's own child of participating in the study; Benefits to the general population; Low risk to the child of participating in the study; Understanding the study and its regulation (percentages per group).<br>Discouraging factors: not mentioned.<br>Other outcomes: factors that improve parents acceptance for consent.<br>Confounding: not mentioned.<br>Level of evidence: B<br>Critical appraisal: Large sample size, not clear what kind of research it consists of, only 4 predefined reasons questioned.
**Wagner, 2006**	Study population: 90 youths and their parents who participated in the clinical treatment research program in child and adolescent psychopharmacology at an academic medical center.<br>Inclusion criteria: not mentioned.<br>Exclusion criteria: not mentioned.<br>Characteristics: children's mean age: 12.37 years (range:6-17), 48% female, 72% Caucasian; parent's mean age: 40.91 years, 82% female, 79% Caucasian; all consenters.<br>Design: Quantitative study; Written pre- and post-study questionnaire to assess attitudes and experiences prior to and upon completion of study.	Motivating factors: Parents: Get treatment for my child 60%, Find out about my child's problem 30%, My child's prior treatment was unsuccessful 5%, Financial reimbursement for visits 2%, Dissatisfied with my child's prior treatment 1%, Treatment is free 1%; Youths: To get help for my problem 43%, To find out what is bothering me: 20%, My parent told me to be in the study: 14%, I will get money when I come here: 11%, To help other people with problems: 4%, My doctor told me to be in the study: 4%, Other: 3%, Treatment is free: 1%.<br>Discouraging factors: not mentioned.<br>Other outcomes: post study questionnaire results.<br>Confounding: not mentioned.<br>Level of evidence: C<br>Critical appraisal: very different drug trials included; people could only give one reason for participation, probably other reasons matter for them also; pre and post questionnaire is a surplus value.

Author, year	Methods	Outcomes
**Wendler, 2012**	Study population: 177 adolescents participating in research at the NIH Clinical Center or Seattle Children's Hospital and their parents.<br>Inclusion criteria: Adolescents 13 to 17 years of age, enrolled in the previous 6 months in a research study for any disorder or as healthy controls at the NIH Clinical Center or Seattle Children's Hospital, spoke English or Spanish, had a parent or guardian who agreed to be interviewed; Parent or guardian of an eligible adolescent who agreed to be interviewed, spoke English or Spanish.<br>Exclusion criteria: when both parents were present, fathers were invited to participate.<br>Characteristics: adolescent's mean age: 15.1 years; 19.8% healthy, 5.1% minor illness, 75.1% significant illness; parent's mean age: 45.3 years; all consenters<br>Design: Quantitative study; personal interviews (questionnaire) with parents and adolescents to conduct an explorative analysis to evaluate whether any of 13 potentially relevant, dichotomized variables were significant.	Motivating factors: "helping find better treatments for others who are ill" is pretty important or very important to their decision to enroll in research (for 84.7% of the adolescents and 87.1% of the parents).<br>Discouraging factors: not mentioned.<br>Other outcomes: willingness to undergo certain procedures.<br>Confounding: not mentioned.<br>Level of evidence: C<br>Critical appraisal: Article focusses on only one reason for participation (helping others), Other reasons were not questioned and explored; researchers do not mention the social desirability of the answer to their main question (helping others); large sample size.
**Woodgate, 2010**	Study population: 31 parents who had a child with a history of cancer at the outpatient paediatric cancer unit at the city's primary cancer treatment center.<br>Inclusion criteria: Ability to speak and understand English; Parents of children with differing cancer diagnoses and at various stages of the treatment completion, from 6 months post diagnosis to 5 years after treatment completion.<br>Exclusion criteria: parents of newly diagnosed cancer patients.<br>Characteristics: parent's age range: 27-51 years; child's age range: 3-17 years; 29 consenters and 2 non-consenters.<br>Design: Qualitative study; person-centered, individual, open-ended interviews. Analyzed with an interpretive descriptive qualitative method (identifying themes).	Motivating factors: doing "the best" for their child (all); the need to help other children with cancer and their families; not disappointing their child's physician.<br>Discouraging factors: not mentioned.<br>Other outcomes: 6 themes identified: living a surreal event (finding it almost an impossible decision to make), wanting the best for my child, helping future families of children with cancer, coming to terms with my decision, making one difficult decision among many, experiencing a sense of trust.<br>Confounding: not mentioned.<br>Level of evidence: +<br>Critical appraisal: Good thing: open-ended question in interview, reasons were not predefined. But no special attention to 2 parents who refused participation in trial and their decision.

Author, year	Methods	Outcomes
**Wynn, 2010**	Study population: 796 parents of infants approached for BABY HUG trial (phase 3 RCT of hydroxyurea)<br>Inclusion criteria: infant <18 months of age, diagnosis of HbSS or HbSb thalassemia.<br>Exclusion criteria: not mentioned.<br>Characteristics: 487 (61%) non-consenters and 309 (39%) consenters.<br>Design: Quantitative study; evaluation of an anonymized registry of potential subjects. Reasons participants stated for decision were categorized in 5 categories.	Motivating factors: Desire to aid research in sickle cell anaemia: 51%; Hope that the child would be randomized to receive hydroxyurea: 51%; Desire to closer follow-up through increased clinic visits: 51%; Perceived the child to be ill and therefore hoped for clinical benefit from participation: 16%.<br>Discouraging factors: high frequency if required clinic visits, blood tests, and special studies: 25%; fear or distrust of research participation: 19%; limited access to transportation: 14%; perceived their child to be healthy and felt medicine was not needed at this time: 10%; wanted their child to receive hydroxyurea rather than possibly being randomized to receive placebo: 2%.<br>Other outcomes: reasons for not approaching.<br>Confounding: classification of responses may have resulted in some misinterpretation of reasons; 21% did not state a reason, could have caused bias.<br>Level of evidence: C<br>Critical appraisal: Good quality: large sample size, prospectively, answers were by free response; Minority group questioned, not generalizable.

Author, year	Methods	Outcomes
**Zupancic, 1997**	Study population: 140 parents who had recently given or declined consent to one of three controlled trials (including drug trial) in the neonatal intensive care unit.<br>Inclusion criteria: not mentioned.<br>Exclusion criteria: Limited English skills.<br>Characteristics: child's median age: 2 days; 103 consenters, 37 non-consenters; no demographic differences.<br>Design: Quantitative study; cross-sectional written questionnaire consisting of 15 socio-demographic items and 13 scaled responses to statements. Responses were subjected to factor analysis to identify underlying constructs. The sample was then randomly split, and multiple regression was performed on each half.	Motivating factors: Factor analysis and multiple regression showed factor: "risk, benefit, and attitudes" to be significantly correlated with consent; consenters had lower parental estimates of risk and higher estimates of benefit, were more likely to report altruistic motives, freedom to make the decision independently and positive attitudes toward research.<br>Discouraging factors: not mentioned.<br>Other outcomes: Factor analysis and multiple regression showed no difference between consenters and non-consenters on "illness severity" or socio-demographic factors.<br>Confounding: not mentioned.<br>Level of evidence: B<br>Critical appraisal: Questionnaire was pretested, had good reliability and validity. Real consent decisions examined; Comparison of consenters and non-consenters; Good response rate.
